# Molecular Mechanisms Involved in the Antitumor Activity of Cannabinoids on Gliomas: Role for Oxidative Stress

**DOI:** 10.3390/cancers2021013

**Published:** 2010-05-26

**Authors:** Paola Massi, Marta Valenti, Marta Solinas, Daniela Parolaro

**Affiliations:** 1 Department of Pharmacology, Chemotherapy and Toxicology, University of Milan, Via Vanvitelli 32, 20129 Milan, Italy; E-Mail: paola.massi@unimi.it; 2 Department of Structural and Functional Biology, Section of Pharmacology, Center of Neuroscience, University of Insubria, Via A. da Giussano 10, 20152 Busto Arsizio, Varese, Italy; E-Mails: marta.valenti@uninsubria.it (M.V.); marta.solinas@uninsubria.it (M.S.)

**Keywords:** cannabinoids, gliomas, apoptosis, cellular mechanism, oxidative stress

## Abstract

Cannabinoids, the active components of *Cannabis sativa*, have been shown to exert antiproliferative and proapoptotic effects on a wide spectrum of tumor cells and tissues. Of interest, cannabinoids have displayed great potency in reducing the growth of glioma tumors, one of the most aggressive CNS tumors, either *in vitro* or in animal experimental models curbing the growth of xenografts generated by subcutaneous or intrathecal injection of glioma cells in immune-deficient mice. Cannabinoids appear to be selective antitumoral agents as they kill glioma cells without affecting the viability of non-transformed cells. This review will summarize the anti-cancer properties that cannabinoids exert on gliomas and discuss their potential action mechanisms that appear complex, involving modulation of multiple key cell signaling pathways and induction of oxidative stress in glioma cells.

## 1. Introduction

In addition to the well-known psychotropic effects of cannabis and its use as an illicit drug, recent studies have suggested a potential application of cannabinoids as therapeutic agents. In particular, cannabinoids, originally derived from the plant *Cannabis sativa*, as well as its endogenous and synthetic counterparts, have been reported to exert antiproliferative actions on a wide spectrum of tumor cells [1,2] and, of relevance, they are able to induce inhibition and regression of gliomas, one of the most aggressive forms of cancer. The present paper will summarize the anti-cancer properties of cannabinoid compounds on gliomas, and discuss their potential action mechanisms with particular emphasis on stress-related cellular effects. 

## 2. Endocannabinoid System

The endocannabinoid system is a recently-discovered signaling system present in both the brain and its periphery, comprising cannabinoid CB_1_ and CB_2_ receptors, their intrinsic lipid ligands endocannabinoids (ECs) such as *N*-arachidonoyl ethanolamide (anandamide, AEA) and 2-arachidonoyl glycerol (2-AG), and associated proteins (transporters and biosynthetic and degradative enzymes). The cannabinoid CB_1_ receptor is a pre-synaptic receptor widely expressed throughout the brain. High densities are present in the striatum, hippocampus, and cerebellum, as well as moderate to low densities in the amygdala, midbrain, and cerebral cortex [3]; it is also present at a lower density in peripheral tissues, including the liver, adipocytes, the exocrine pancreas, the GI tract, skeletal muscle and circulating immune cells [4].

CB_2_ receptors were cloned a few years after CB_1_ [5], and while they were thought to be predominately located in immune cells in tissues such as the spleen and the liver, there is recent evidence that cannabinoid CB_2_ receptors exhibit limited neuronal expression [6,7,8,9,10,11]. 

Both CB_1_ and CB_2_ are G-protein coupled receptors. The CB_1_ receptor couples with both Gi/o proteins which function to inhibit adenylyl cyclase activity, activate potassium channels and inhibit voltage-gated calcium channels, while the CB_2_ receptor is only known to couple with Gi proteins [12]. The discovery of endogenous ligands for the cannabinoid receptor (endocannabinoids) occurred soon after the characterization of the receptor. The two primary ligands which have been characterized as endocannabinoid are *N*‑arachidonoylethanolamine, or anandamide (AEA) and 2-arachidonoylglycerol. Both AEA and 2-AG are formed post-synaptically from phospholipid precursors through activity-dependent activation of specific phospholipase enzymes [13] 

Endogenous ligands do not share the same biosynthetic or metabolic pathways, indicating distinct regulation mechanisms. Multiple biochemical pathways may synthesize AEA [14] and the primary pathway for the production of AEA within the CNS has not been clearly determined yet. 2-AG is mainly synthesized through activation of phospholipase C, and the subsequent production of diacylglycerol, which is rapidly converted to 2-AG by diacylglycerol lipase [15]. AEA is hydrolyzed by the enzyme fatty acid amide hydrolase (FAAH), generating arachidonic acid and ethanolamine, while 2-AG is primarily metabolized by monoacylglycerol lipase (MAG lipase), which results in the formation of arachidonic acid and glycerol [16]. The presence of two endogenous ligands for one receptor has not been fully explained, but differences in pharmacokinetics and in the efficacy of these ligands has been demonstrated [17], suggesting that they may play distinct physiological roles. Furthermore, endocannabinoid signaling acts differently than most neurotransmitter systems. Specifically, endocannabinoids are released “on demand” by post-synaptic cells, function as retrograde signals and traverse back across the synapse where they bind with pre-synaptically located CB_1_ receptors and reduce synaptic transmitter release [16].

Gliomas have been shown to possess one or more components of the endocannabinoid system such as the ability to synthesize endocannabinoids, the presence of CB_1_/CB_2_ receptors and the enzyme FAAH, thus suggesting a possible role of this system in regulation of cell growth.

## 3. Gliomas and Cannabinoids

Malignant gliomas are the most common type of brain tumor in adults, and high-grade gliomas (glioblastomas, GBMs) are among the most rapidly growing and devastating neoplasms. GBMs rarely metastasize out of the central nervous system, but their aggressive invasion of normal tissue surrounding the tumor mass makes surgical removal virtually impossible, and substantially complicates clinical management of the disease [18,19]. Despite surgery and radiotherapy, these tumors invariably recur, and generally lead to death within less than one year of diagnosis. 

A hallmark characteristic of gliomas is their molecular and cellular heterogeneity (either in terms of pathology or genetic changes even within a single tumor) which is considered one of the reasons for their malignancy [20,21]. A large number of chemotherapeutic agents (e.g., alkylating agents such as temozolomide, and nitrosureas such as carmustine) have been tested, but no remarkable improvement in patient survival has been achieved so far. Therapeutic adjuvant to surgical resection such as focal radiotherapy and chemotherapy provide only a negligible improvement in the disease’s course and life expectancy with a variable toxicity profile among these treatments, with myelosuppression being the most frequent and limiting factor. Despite this multimodality treatment, clinical recurrence or progression is nearly universal and available systemic chemotherapies offer only modest clinical benefits. Likewise, although immunotherapy strategies appear promising as a new and safe approach to induce antitumor immune response [22], no immunotherapy or gene therapy trial performed to date has been significantly successful.

## 4. Δ^9^-Tetrahydrocannabinol and Glioma Growth

In 1998, Guzman’s group reported the antitumoral effect of Δ^9^-tetrahydrocannabinol (THC) in C6 murine glioma cells [23]. It was shown that THC-induced glioma cell death was independent of CB_1_ cannabinoid receptor stimulation and accompanied by a significant breakdown of cellular sphingomyelin pathways [24]. Given the favorable safety profile, in March 2002, the Spanish Ministry of Health approved a Phase I/II clinical trial, carried out in collaboration with Tenerife University Hospital and Guzman’s research group, aimed at investigating the effect of local administration of THC on the growth of recurrent glioblastoma multiforme. The study was the first pilot study that investigated cannabinoid antitumoral action but also the intracranial application of THC through an infusion cannula connected to a subcutaneous reservoir. The nine enrolled patients had previously failed standard therapy (surgery and radiotherapy) and constituted a cohort of terminal patients harboring actively-growing recurrent tumors. The results have been recently published [25]. THC delivery was safe and achieved without overt psychoactive effects. Median survival of the cohort from the beginning of cannabinoid administration was 24 weeks, but two patients survived for approximately one year. Survival for GBM patients following diagnosis is typically six to 12 months. The authors reported that due to the characteristics of the study, the effect of THC on patient survival was unclear, and evaluation of survival would require a larger trial with a different design. However, in placebo-controlled trials for recurrent glioblastoma multiforme with temozolomide, a slight impact has been reported on overall length of survival (median survival: 24 weeks, 6-month survival = 46–60%).

Thus, the possibility to have other drugs such as cannabinoids available to manage these devastating tumors has improved research to establish their action mechanisms on tumor cells and to definitively establish their efficacy. Studies with animal models have shown that local administration of THC or the synthetic cannabinoid WIN-55,212-2 reduced *in vivo* the size of the tumor generated by intracranial inoculation of C6-derived glioma in Wistar rats [26] with a concomitant involvement of CB_1_ and CB_2_ receptors. Moreover, rats bearing malignant gliomas, when treated intratumorally with cannabinoids, survived significantly longer than untreated animals and 20–35% of treated animals showed a complete eradication of the tumors.

## 5. CB_2_ Selective Compounds and Glioma Growth

The unwanted psychotropic effects of marijuana-derived cannabinoids are mediated largely or completely by neuronal CB_1_ receptors. Thus, great efforts have been made to assess alternative possibilities. One of the most obvious strategies to avoid psychotropic side effects in the management of glioma tumor growth is the administration of CB_2_-selective compounds. Recent evidence that CB_2_ receptors are present in both cultured neurons and the nervous system has to be taken into account [27,28]. The co-expression of the CB_1_ and CB_2_ cannabinoid receptors has been detected in rat C6 glioma cells and in biopsies from human astrocytomas [29]. Moreover, the extent of CB_2_ expression was related to tumor grade. Another study that surveyed the level of CB_2_ receptors in biopsies of human astrocytomas and glioblastomas revealed a high level of this receptor subtype among adult and pediatric tumors [30] and its amounts appeared to be correlated with tumor malignancy. Calatazzolo *et al*. [31] found a higher expression of CB_2_ receptors in glioblastomas as well as in endothelial cells than in low-grade gliomas. High levels of CB_2_ expression in either gliomas or in endothelial cells of glioblastoma vessels was also demonstrated by Schley *et al*. [32]. High levels of CB_2_ expression suggest that these tumors would be vulnerable to a cannabinoid treatment and indicate a specific CB_2_ cannabinoid agonist-based strategy. In this context, it has been demonstrated that the local and *in vivo* daily administration of the selective CB_2_ agonist JWH-133 in mice bearing subcutaneous glioma causes a considerable regression of malignant tumors, inducing a classic pattern of apoptosis via ceramide de novo synthesis [29].

The hypothesis of the usage of CB_2_ selective compounds has prompted further research on the effectiveness of a series of novel CB_2_ cannabinoid compounds in glioma treatment [33]. The lead compound named KM-233 represents the first generation of synthetic C1’ aryl substitute cannabinoid ligands. This compound exhibits a good lipophilicity and affinity for the CB_2_ receptor that could predict significant transit across the blood brain barrier and good activity at the CB_2_ receptor on glioma cells. KM-233 has shown excellent cyotoxicity against U87, U373 and C6 glioma cells and it is also effective *in vivo* in reducing glioma tumor growth subcutaneously in SCID mice, via both direct intra-tumoral injection and systemic administration. In addition, another series of CB_2_ selective synthetic compounds has been tested in human glioma cells and found to be highly cytotoxic to cells [34]. Of particular interest, Aguado *et al*. demonstrated the inhibition of gliomagenesis induced by the selective CB_2_ compound JWH-133 on glioma stem-like cells [35].

## 6. Non Psychotropic Cannabinoids and Glioma Growth

Another strategic approach that has been pursued is to explore the usage of natural, non-psychotropic cannabinoids that bind with very low affinity to cannabinoid receptors, thus excluding either psychotropic and/or immune/peripheral effects. Among the bioactive constituents of marijuana, cannabidiol (CBD), does not have significant intrinsic activity on cannabinoid receptors [28,36] and does not induce psychotropic and adverse side effects. For these reasons, it is one of the natural cannabinoids with the highest potential for therapeutic use. A first study of Massi *et al*. [37] reported that CBD was effective in inhibiting U87 and U373 human glioma cell proliferation in an *in vitro* set of experiments. Additional experiments demonstrated *in vivo* the antitumor activity of CBD [37]. When tumor xenografts generated by subcutaneous injection of glioma cells in the flank region of immune-deficient mice were treated locally with CBD, there was a significant 60% mean reduction of tumor growth over a 23-day period of observation, although no eradication was described [37]. The antiproliferative effect of CBD was dose-correlated and dependent on its ability to induce apoptotic death. All these effects appeared independent of cannabinoid receptor stimulation [37]. Bisogno *et al.* [38] have reported that cannabidiol can recognize the transient receptor potential vanilloid type-1 (TRPV1) as a molecular target, demonstrating that the drug is a full, although weak, agonist on human TRPV1. Ligresti *et al.* [39] showed that in addition to cannabidiol, the plant cannabinoids cannabigerol and cannabidiol acid were found to activate TRPV1 receptors. Thus, it can be suggested that there are some alternative ways through which nonpsychotropic cannabinoids can induce apoptosis since it is possible that when TRPV1 is stimulated, apoptosis may be induced by mitochondrial events triggered by TRPV1-mediated calcium influx [40]. Recently, De Petrocellis *et al.* [41] have also demonstrated an interaction of phytocannabinoids with ankyrin TRPA1 and melastatin TRPM8 channels, with potential implications for the cancer treatment.

The capability of cannabidiol to either potentiate or inhibit the actions of THC was recently examined by the McAllister group. In the U251 and SF126 glioblastoma cell lines, THC and CBD acted synergistically to inhibit cell proliferation [42]. The treatment of glioblastoma cells with both compounds led to a significant modulation of the cell cycle, induction of reactive oxygen species and apoptosis as well as specific modulations of extracellular signal-regulated kinase and caspase activities. These specific changes were not observed with either compound individually, indicating that the signal transduction pathways affected by the combination treatment were unique. These results suggest that the addition of CBD to THC may improve the overall effectiveness of THC in the treatment of glioblastoma in cancer patients. 

Finally, the synthetic derivative of THC, ajulemic acid, has also been reported to inhibit glioma cell growth *in vitro* and *in vivo* inducing cytostatic rather than cytotoxic effects [43], although its pharmacological properties are still controversial and not completely clarified.

## 7. Endocannabinoids and Glioma Growth

Ongoing research is now evaluating whether endogenous cannabinoids exert tumor-suppressing effects in glioma growth, thus potentially representing an alternative approach to the development of possibly harmless anti-cancer drugs [44,45]. In fact, endogenous cannabinoid agonists or selective inhibitors of endocannabinoid degradation with limited action on CB_1_ receptors, would exhibit little if any psychotropic activity and be effective only in those tissues where the levels of endocannabinoids were altered. However, the anti-tumor potential of substances that modulate the endocannabinoid system is still largely unexplored. 

The use of AEA would have a number of additional advantages over THC: (a) AEA is virtually ineffective on CB_2_ receptors, which would rule out the immunosuppressive effect described for THC. (b) AEA has been shown to promote the growth of hematopoietic cell lines, an effect which may be particularly attractive if AEA-enhancing strategies were to be included in polychemotherapeutic protocols. By contrast, the poor stability and short half-life of AEA make its use as a therapeutic agent largely impractical. However, since a number of tumor cell lines express one or more components of the endocannabinoid system and since AEA is synthesized on demand at multiple sites throughout the body and because of its lipophilic feature it easily reaches tumor sites as well, including the CNS [45], novel antiproliferative strategies based on the pharmacological modulation of AEA levels through inhibition of AEA uptake and/or degradation by FAAH (an approach which would interfere with endocannabinoid levels mildly and in a neuronal activity-dependent fashion) may be considered for the clinical management of at least some forms of neoplastic disease. 

Finally, the studies on the putative anti-tumor properties of endogenous cannabinoids in human gliomas are only beginning. It has been demonstrated that AEA induces apoptosis in cells derived from the neural crest, such as the CPH100 human neuroblastoma cell line through a pathway involving a rise in intracellular calcium, mitochondrial uncoupling and cytochrome c release [40]. Unlike AEA, other ECs such as 2-AG, linoleoylethanolamide (LEA), oleoylethanolamide (OEA), and palmitoylethanolamide (PEA) were unable to force cells into death [40]. Jacobsson *et al*. [46] showed that in rat C6 glioma cells AEA exerts antiproliferative effects associated with a combined activation of cannabinoid and vanilloid receptors but, in contrast with Maccarrone’s data [40], 2-AG inhibited glioma cell proliferation with a similar potency to that of AEA. Another EC such as stearoylethanolamide (SEA), present in the human brain in amounts comparable to those of AEA, induced pro-apoptotic activity in glioma cell line [47]. Contassot *et al*. [48] showed that human glioma cell lines, either established for a very long time (U87 and U251) or derived from a tumor biopsy (Ge227 and Ge258) are efficiently killed by AEA. These cell lines contemporarily express CB_1_, CB_2_ and the transient receptor potential vanilloid type-1 (TRPV1), and the authors demonstrated that the antiproliferative effects of AEA were essentially due to its ability to bind to the TRPV1 receptor. The stimulation of the TRPV1 receptor by endocannabinoids could represent an alternative mechanism through which AEA causes apoptosis triggering calcium influx in tumor cells [40]. Despite the scarce data available, the selective targeting of TRPV1 and/or CB_1_/CB_2_ receptors by EC system modulation could represent an attractive area of drug development, avoiding CB_1_-mediated psychotropic side effects and CB_2_-mediated immunosuppression. In addition, another study has demonstrated that the commonly used acylderived AEA uptake inhibitors AM404, VDM11, UCM707 and OMDM2 rapidly affected C6 glioma cell viability [49].

## 8. Cellular Action Mechanisms of Cannabinoids on Gliomas and Cells Derived from the CNS

Natural, synthetic and endogenous cannabinoids have all been found to affect a number of pathways involved in the cell survival/death decision in cell lines derived from the CNS and progress has been made towards understanding the intracellular mechanisms underlying *in vivo* and *in vitro* antitumor effects.

The signaling pathways activated by cannabinoids to induce tumor cell death have been studied in primary astrocytes and rat and human glioma cell lines, as well as in CB_1_-transfected CHO cells and a number of downstream effectors have been identified.

The apoptosis induced by THC in C6 cells is accompanied by intracellular accumulation of the ubiquitous lipid second messenger ceramide and by activation of one or more families of mitogen-activated protein kinases (MAPKs) [50]. Following exposure to THC, a biphasic pattern of ceramide accumulation has been observed in C6 rat glioma cells, with an early peak occurring within minutes, followed by a sustained second generation of ceramide lasting several days [51,52]. This delayed peak of ceramide generation has been proposed to be fundamental for apoptosis and depend on *de novo* ceramide synthesis, rather than on sphingomyelin hydrolysis. The close relationship between ceramide accumulation and THC-induced apoptosis is also supported by the finding that primary neurons are resistant to cannabinoid-induced ceramide accumulation and apoptosis, unless fairly high THC concentrations are used [53,54].

Moreover, Carracedo *et al.* [24], using wide array of experimental approaches, identified the stress regulated protein p8 as an essential mediator of cannabinoid antitumoral action and showed that p8 up-regulation is dependent on de novo-synthesized ceramide [24]. The p8 upregulation also takes place *in vivo* and resistance to cannabinoid treatment is associated with a decreased activation of the p8-regulated proapoptotic pathway [24].

The p8 target is the pseudokinase tribbles homolog 3 (TRB3) and recently the mechanism that promotes the activation of this signaling route as well as the target downstream of TRB3 that mediates its tumor cell-killing action has been partially elucidated. In fact Salazar *et al*. [55] showed that in human glioma cells THC-induced ceramide accumulation and the eukaryotic translation initiation factor 2α (eIF2α) phosphorylation thereby activating an ER stress response that promoted autophagy via tribbles homolog 3–dependent (TRB3-dependent) inhibition of the Akt/mammalian target of rapamycin complex 1 (mTORC1) axis [55]. The autophagy is upstream of apoptosis in THC-induced human and mouse cancer cell death and the activation of this process was necessary for the antitumor effects of cannabinoids *in vivo*. 

THC has been demonstrated to acutely enhance the activity of MAPKs, particularly ERKs and JNKs (Jun *N*-terminal kinases) in a CB_1_ receptor-dependent fashion, with a time course which parallels that observed for ceramide accumulation [50,51]. However, the relationship between ceramide accumulation and MAPK activation following CB_1_ receptor engagement by THC is far from obvious, as an increase in intracellular ceramide levels does not seem to be a prerequisite for cannabinoid-induced activation of the JNK family of MAPKs [52]. 

Mechanisms other than ceramide induction can be involved in cannabinoid-induced cell death. Massi *et al*. demonstrated that there was no involvement of ceramide in CBD-induced apoptosis [56], thus suggesting that CBD and/or other cannabinoids can exert their antineoplastic effects independently by stimulation of this downstream effector. 

A report discloses that WIN inhibits C6 glioma cell proliferation through an inhibition of ERK1/2 kinase and AKT, the key mediator of growth factor-promoted cell survival. A decrease of mitogenic/pro-survival signaling precedes reduction of Bad phosphorylation and the events that follow Bad translocation to the mitochondrial membrane. Bad, a pro-apoptotic Bcl2 family member, may be an important link between the down regulation of the survival pathway and caspase activation evoked by cannabinoid treatment and resulting in glioma cell death [57].

An involvement of PI3K in the acute effects of CB_1_ receptor stimulation has also emerged from a study on CB_1_-transfected CHO cells and CB_1_-expressing human U373 MG astrocytoma cells, where THC has been shown to enhance the activity of protein kinase B (PKB)/Akt [58]. PKB plays a fundamental role in the regulation of basic cell functions, such as energy metabolism and proliferation. 

Finally, evidence has been collected supporting the role of cannabinoids in controlling glioma cell growth through the inhibition of lipoxygenase (LOX)-enzyme. *In vivo* treatment of nude mice bearing subcutaneous glioma tumors with CBD was found to inhibit the activity and content of 5-LOX enzyme in tumor tissues by a significant 40% [59]. 

All this data provides further demonstration of other and/or alternative intracellular targets that can be importantly modulated by cannabinoids contributing to their evident antitumoral effects.

## 9. Role of Oxidative Stress in the Antiproliferative Effects of Cannabinoids

Under physiological conditions, the maintenance of an appropriate level of intracellular reactive oxygen species (ROS) is important in keeping redox balance and standard signaling proliferation. ROS are essential for many biological functions. They can regulate many signal transduction pathways by directly reacting with and modifying the structure of important protein transcription factors and genes. The modulation of their function can ultimately alter the expression and activities of many transcription factors as well as signaling proteins that are involved in the stress response and cell survival through multiple mechanisms. Moreover, an overproduction of ROS or decreased ability to scavenge would result in a significant increase of intracellular ROS, leading to cellular damage, lipid peroxidation, DNA modifications and enzyme inactivation. If the ROS level is consistent and persistent, all of these damages can cause cell death. It is well-known that cancer cells are characterized, in general, by high levels of ROS and intrinsic oxidative stress. Compared with normal cells, cancer cells seem to possess higher levels of endogenous ROS but events that increase ROS levels above a certain threshold seem to induce an incompatible situation with cellular survival, leading to cell death. This provides the rationale for killing cancer cells inducing ROS accumulation in malignant cells with appropriate agents. Thus, treating cancer cells with compounds that possess pro-oxidant properties and increase ROS level or that abrogate the cellular antioxidant system will shift the redox balance resulting in cancer cell cytotoxicity. 

Besides the above-mentioned molecular mechanisms underlying antitumoral action of cannabinoids, evidence has been collected showing that an additional cellular mechanism through which cannabinoids can modulate cell survival/death fate is the induction of oxidative stress in cancer cells. Jacobsson *et al*. [46] showed that AEA and 2-AG produce antiproliferative effects on rat C6 glioma cells by a mechanism that involves both cannabinoids and vanilloid receptors and oxidative stress since the anti-oxidant α-tocopherol completely reversed the antitumoral activity of the cannabinoids compounds either at 0.1 μM or 10 μM concentration. 

Goncharov *et al*. [60] reported that the activation of CB_1_ receptors by THC in C6 cells makes these cells more vulnerable to oxidative damage. The study was performed using a cell permeating Fe (III) chelating quinone that provided more physiological conditions for mimicking naturally occurring oxidative stress within the cell, as a better model for natural ROS formation. In fact, the addition of THC for 10 min prior to the induction of oxidative stress increased subsequent cell damage as demonstrated by LDH and MTT assay. This effect was reversed by the addition of the CB_1_ but not the CB_2_ selective antagonist. The authors also reported a parallel decrease in glucose uptake, probably contributing to a dramatic depletion in the energy reserve of the c ells. This event could render the gliomas cells more sensitive to oxidative stress, driving them into apoptosis. Massi *et al*. [37] also demonstrated that the pretreatment of glioma cells with α-tocopherol antagonized the anti-proliferative effect of the non psychotropic cannabinoid compound CBD, thus suggesting an involvement of oxidative stress in CBD-antitumoral effects. The authors then investigated the presence of the existence of an oxidative stress state in gliomas cells after CBD exposure [61]. They found that CBD induced significant ROS production, GSH depletion and increase activity of GPox and GRed enzymes, as early as 5–6 h after CBD exposure, with a time course preceding caspases activation. The authors concluded that when the generation of ROS exceeds the scavenging capacity of the cell, and if there is a contemporary decrease in GSH levels counteracted by the increased activity of associated anti-oxidant enzymes, the cell could initiate cell death-linked molecular events, namely the activation of caspase-9 and -8 which, in turn, cleave caspase-3. Ligresti *et al.* [39] demonstrated that the antiproliferative action of 10 μM CBD in MDA-MB-231 breast cancer cells was significantly prevented by the antioxidant tocopherol, vitamin C as well as astaxantine. They showed that CBD induced ROS formation and that this effect was Ca^2+^ dependent because it was erased when cells were preloaded with the Ca^2+^ chelator BAPTA-AM. The rise of intracellular ROS production can account for the proapoptotic effects of CBD in tumor cells although the phenolic chemical structure would rather favor an antioxidant effect. The oxidant properties of CBD can be dependent from the different biochemical and cellular features of tumor versus non-tumor cells rather than from the molecule itself [61] and/or by its ability to cause an increase in intracellular Ca^2+^ depending on the cell culturing conditions [39].

Marcu *et al*. [42] demonstrated on human glioblastoma cells that the combination of THC and CBD produced a significant increase in the formation of ROS. The observed initial increase in ROS was clearly linked to a latter induction of apoptosis. Individually, both THC and CBD could increase apoptosis through the production of ROS, but THC was significantly less efficient at inducing this process as a single agent as compared with when it was used in combination with CBD. Although the concentration of CBD used in the combination treatment did not significantly stimulate ROS, it may have primed this pathway for THC through a convergence on shared signal transduction pathways.

The induction of oxidative stress induced by cannabinoids was also a common mechanism reported in other types of cancer cells since either in EL-4 thymoma [62], in human colorectal carcinoma Caco-2 [63], and in PC-12 cells [64] it was demonstrated that oxidative stress plays a role in the antiproliferative effect of cannabinoids. 

## 10. Conclusions

The therapy of gliomas, the most frequent class of malignant primary brain tumors and one of the most aggressive forms of cancer characterized by high invasiveness, a high proliferation rate and rich neovascularization, could benefit from the use of cannabinoids, the active compounds of *Cannabis sativa*, and their synthetic derivatives. They have been shown to mimic the endogenous substances named “endocannabinoids” that activate specific cannabinoid receptors (CB_1_ and CB_2_).

Cannabinoids have been proven to inhibit glioma tumor growth in either *in vitro* or *in vivo* models through several cellular pathways such as elevating ceramide levels, modulating PI3K/Akt, MAPK kinases, inducing autophagy and oxidative stress state in glioma cells, thus arresting cell proliferation and inducing apoptosis. Since cannabinoids kill tumor cells without toxicity on their non transformed counterparts, probably modulating the cell survival/cell death pathways differently, they can represent a class of new potential anticancer drugs. 
